# Homozygous *STIL* Mutation Causes Holoprosencephaly and Microcephaly in Two Siblings

**DOI:** 10.1371/journal.pone.0117418

**Published:** 2015-02-06

**Authors:** Charlotte Mouden, Marie de Tayrac, Christèle Dubourg, Sophie Rose, Wilfrid Carré, Houda Hamdi-Rozé, Marie-Claude Babron, Linda Akloul, Bénédicte Héron-Longe, Sylvie Odent, Valérie Dupé, Régis Giet, Véronique David

**Affiliations:** 1 Institut de Génétique et Développement de Rennes, Equipe Génétique des Pathologies Liées au Développement, Faculté de Médecine, Université de Rennes 1, 35043 Rennes, France; 2 Institut de Génétique et Développement de Rennes, Equipe Cytosquelette et Prolifération Cellulaire, Faculté de Médecine, Université de Rennes 1, 35043 Rennes, France; 3 Laboratoire de Génétique Moléculaire et Génomique, CHU Pontchaillou, 35033 Rennes, France; 4 Inserm U946, Variabilité Génétique et Maladies Humaines, Université Paris-Diderot, 75010 Paris, France; 5 Service de Génétique Clinique, Hôpital Sud, 35200 Rennes, France; 6 Service de Pédiatrie, Hôpital Jean Verdier, APHP, 93140 Bondy, France; 7 Plateforme Génomique Santé, Biosit, Université Rennes 1, 35033 Rennes, France; Icahn School of Medicine at Mount Sinai, UNITED STATES

## Abstract

Holoprosencephaly (HPE) is a frequent congenital malformation of the brain characterized by impaired forebrain cleavage and midline facial anomalies. Heterozygous mutations in 14 genes have been identified in HPE patients that account for only 30% of HPE cases, suggesting the existence of other HPE genes. Data from homozygosity mapping and whole-exome sequencing in a consanguineous Turkish family were combined to identify a homozygous missense mutation (c.2150G>A; p.Gly717Glu) in *STIL*, common to the two affected children. *STIL* has a role in centriole formation and has previously been described in rare cases of microcephaly. Rescue experiments in U2OS cells showed that the *STIL* p.Gly717Glu mutation was not able to fully restore the centriole duplication failure following depletion of endogenous STIL protein indicating the deleterious role of the mutation. *In situ* hybridization experiments using chick embryos demonstrated that expression of *Stil* was in accordance with a function during early patterning of the forebrain. It is only the second time that a *STIL* homozygous mutation causing a recessive form of HPE was reported. This result also supports the genetic heterogeneity of HPE and increases the panel of genes to be tested for HPE diagnosis.

## Introduction

Holoprosencephaly (HPE) (#236100) is the most frequent congenital malformation of the brain (1 in 10,000 live births; 1 in 250 conceptuses). HPE is characterized by impaired forebrain cleavage, midline facial anomalies and wide phenotypic spectrum. The clinical spectrum ranges from alobar HPE to semilobar and lobar HPE generally associated with facial anomalies [1]. HPE is a severe pathology with mental retardation and developmental delay in all affected live newborns, with poor or symptomatic treatment. In addition, the midline malformation affects the development of the hypothalamus and the pituitary gland, leading to frequent endocrine disorders like temperature, heart rate and respiration instabilities, hypogonadism, thyroid hypoplasia or diabetes insipidus. The oromotor dysfunction is also affected, with feeding and swallowing difficulties [2]. Only 20% of children with alobar HPE survive after the first year of life, and 50% of infants with semi-lobar HPE are alive after 12 months [3]. Isolated HPE presents a high genetic heterogeneity and to date heterozygous mutations in 14 genes have been identified in HPE patients, 4 major genes (*Sonic hedgehog* or *SHH*, *ZIC2*, *SIX3*, *TGIF1*) and 10 genes considered as minor genes *(PTCH1*, *TDGF1*, *FAST1*, *GLI2*, *DISP1*, *FGF8*, *GAS1*, *CDON*, *NODAL* and *DLL1*) [4–7]. These genes encode proteins playing a role in early development, belonging mostly to signaling pathways like Sonic Hedgehog (SHH) or Nodal [8]. However, many patients remain without a molecularly confirmed diagnosis. In 70% of isolated cases, mutations in *SHH*, *SIX3 and TGIF1* are inherited from a parent unaffected or harboring a microform of HPE [1], suggesting that other events are necessary to develop the disease. Thus, the mode of inheritance described as autosomal dominant with an incomplete penetrance and a variable expression has evolved to the assumption of a multi-hit pathology requiring two or more events involving several genes from the same or different signaling pathways.

The existence of rare consanguineous families suggests the possibility that autosomal recessive inheritance may account for a substantial part of this disorder. In the case of a rare recessively inherited disorder and known consanguinity, the initial assumptions are that the disease is caused by a homozygous variant inherited from both parents and that this variant resides within a large homozygous region.

**Fig 1:**
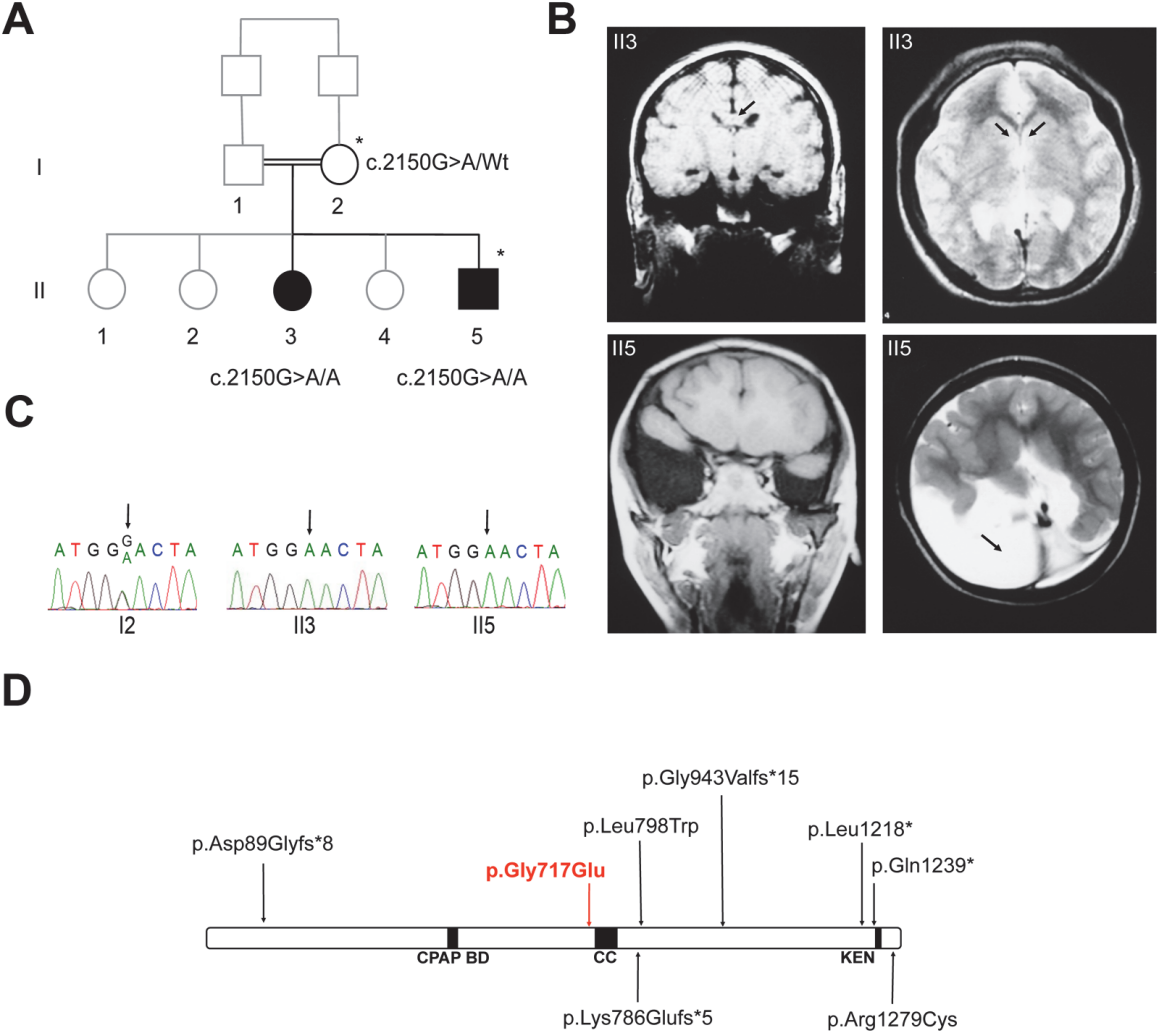
Pedigree of the consanguineous family, brain MRI of the affected siblings, Sanger validation of the c.2150G>A (p.Gly717Glu) *STIL* mutation, and schematic report of all *STIL* mutations reported so far. (A) Pedigree of the inbred family. Closed symbols indicate individuals affected with holoprosencephaly. Family members marked with an asterisk were analyzed by whole exome sequencing. (B) Coronal (on the left) and axial (on the right) brain MRI in individuals II3 and II5 at 12 and 5 years old respectively. II3: lobar HPE, the arrow in the coronal section shows the corpus callosum, and the arrows in the axial section show the absence of visualization of frontal horns, and a partial agenesis of the corpus callosum; II5: semi-lobar HPE, the arrow on axial MRI shows the absence of occipital lobe and a large unilateral temporal and occipital fluid cavity communicating. (C) Sanger validation was performed for the 3 available individuals I2, II3 and II5. The c.2150G>A mutation in *STIL* revealed a segregation with HPE in the two affected children. (D) Distribution of mutations previously reported in the literature on STIL protein. All mutations were present in a homozygous state [21,23,29] except those represented under the protein, which were two compound heterozygous mutations [28]. The p.Leu1218* mutation was found twice in two different families. The mutation reported in this study is p.Gly717Glu (in red) and is located in the central domain of the protein. Three important domains were represented here, the CPAP binding domain from amino acid 429 to 448, the coiled-coil domain (CC) from amino acid 720 to 750 and the KEN box, located between amino acids 1243–1245.

To test this hypothesis and to improve the genetic basis of HPE, we have employed homozygosity mapping in two affected siblings and their mother issued from a Turkish consanguineous family (parents were first cousins), coupled with a next-generation sequencing approach on DNA from the mother and only one of the affected siblings (Fig. 1A). DNA of the father was not available.

## Material and Methods

All patient samples in this study were obtained with informed consent according to the protocols approved by the local ethics committee (Rennes hospital).

### Homozygosity Mapping

Genome-wide genotyping was undertaken using Illumina 300K single nucleotide polymorphism (SNP) mapping array beadchip (Illumina), on DNA of individuals I2 (mother), II3 (sibling—girl) and II5 (sibling—boy). The BlueFuse Multi software v3.3 (BlueGnome) was used to identify homozygous regions in all the family members. Only the regions longer than 1Mb and carrying at least 100 consecutive homozygous SNPs were selected. In parallel, homozygous regions and inbreeding coefficients were estimated/analyzed using FSuite pipeline [9].

### Whole exome sequencing and variants filtering

Exome sequencing was performed on DNA of the boy II5 and of the mother I2 by Integragen using SureSelect V5 capture kit (Agilent) on HiSeq system (Illumina). The mean coverage was 80x, 95% of sequences had at least 10-times coverage and 91% of sequenced bases had a quality score greater than or equal to Q30. Bioinformatics analysis has been conducted with the Illumina pipeline analysis CASAVA v1.8. The sequenced reads were aligned to the hg19 human reference genome with Eland v2 before variant calling (SNV and INDEL) with the CASAVA suite. The variants were then integrated into the Integragen proprietary online tool ERIS v2.0 (http://eris.integragen.com/) and filtered according to their genotype, allele frequency, and variant position within the gene. Based on the assumption that the mutation underlying the HPE was recessive in this inbred family, only mutations at a homozygous state in the boy (ll5) and at a heterozygous state in the mother (l2) were retained. Resulting variants were annotated using a February 2014 build of ANNOVAR [10]. This software maps variants to RefSeq genes, known variations and frequencies from dbSNP137; it also annotates the predicted functional consequences of missense variants using six prediction algorithms (SIFT, Polyphen2, LRT, Mutation Taster, Mutation Assessor and FATHMM) and three conservation scores (PhyloP, GERP++ and SiPhy) from the dbNSFP v2.0 [11]. Predictions by two new ANNOVAR in-house prediction scores were also performed. These algorithms, MetaSVM and MetaLR, use a radial Support Vector Machine (SVM) model that is based on multiple other scores, and according to the ANNOVAR website, the model outperforms other algorithms (developed by Coco Dong and Dr. Xiaoming Liu, University of Southern California, http://www.openbioinformatics.org/annovar/annovar_filter.html). Complementary annotations were performed using Condel v2.0 [12], Alamut v2.3 (Interactive Biosoftware) and Ingenuity Variant Analysis (www.ingenuity.com/variants) softwares.

### Sanger sequencing

The *STIL* mutation c.2150G>A was confirmed in the mother I2 and the boy II5, and searched for in the girl II3 by classic Sanger sequencing. This was done using the BigDye terminator cycle sequencing kit (Applied Biosystems) on an ABI3130xl sequencer (Applied Biosystems) and analyzed using SeqScape software v2.6 (Life Technologies).

### Centriole duplication assay

The plasmid expressing GFP-STIL p.Gly717Glu was obtained by site-directed mutagenesis from a pEGFP-C1 plasmid containing cDNA of GFP-STIL, kindly provided by Alwin Kramer (German Cancer Research Center, Heidelberg, Germany). These plasmids were resistant to the siRNA used for depletion of endogenous STIL. The sequence of the siRNA used was 5’-CAGUAACUCUAGCAAAUAA-3’. U2OS cells (human osteosarcomas cells) were grown at 37°C in DMEM media containing 10% fetal calf serum and 100 U/ml of streptomycin and penicillin. Rescue experiments were performed as described in Franck *et al* [13]. Cells were transfected using JetPRIME transfection reagent (Polyplus Transfection), first with 100 pmol/ml of siRNA, and 24h later with 600 ng/ml of plasmids pEGFP-STIL WT or pEGFP-STIL p.Gly717Glu. At the same time, cells were synchronized in G1/S phase with aphidicolin at 4 μl/ml. Cells were fixed 36h after plasmid transfection with methanol. For counting, centrioles were labeled using a mouse anti-centrin antibody (Millipore, clone 20H5) and GFP-STIL was labeled using a rabbit anti-GFP antibody (Abcam, ab-89314). The experiments were repeated at least three time and a minimum of 50 cells were counted for each experiment.

### *In Situ* Hybridization

Fertilized chicken (*Gallus gallus*) eggs were obtained from EARL Les Bruyères (France). For the required developmental stages Hamburger and Hamilton (HH) 8, HH10 and HH16, eggs were incubated in a humidified incubator at 38°C [14]. After harvesting, chick embryos were fixed with 4% paraformaldehyde in phosphate buffered saline. The *Stil* probe was generated by PCR using the *Gallus gallus* NCBI *Stil* sequence, and subcloned in the pCRII-TOPO vector (Invitrogen, Cergy Pontoise, France). The recombinant plasmid was used to transcribe the sense and antisense RNA probes, labeled with digoxygenin. Whole-mount *in situ* hybridization was performed as previously described [15].

## Results and Discussion

The two HPE affected children (born alive) and their three healthy siblings were seen in France, the girl (II3) was 12 years of age and the boy (II5) was 5 years of age (Fig. 1A, B).

For II3, her weight was 21.7 kg (-2.5 Standard Deviation, SD), height 1.24 m (-3SD), and occipitofrontal circumference (OFC) 41 cm (microcephaly <-7SD). Although the OFC was not recorded at birth, microcephaly was reported to be congenital. She had a severe intellectual disability, said only a few words, with a good understanding. Her walk was normal and she had no behavioral or sleep defects. Brain MRI showed lobar HPE, absence of ventricular frontal horns, partial agenesis of the corpus callosum, both anterior and of the splenium (Fig. 1B).

For II5, his weight was 14 kg (-2SD), height 1 m (-2SD), and OFC 41.5 cm (microcephaly <-8SD). Microcephaly was also congenital. He had hypotelorism, major behavioral disorders, with episodes of self-aggression and sleep disorders. He had no language or acquired sphincter control. His walk was normal. He developed generalized tonic clonic seizures. Brain MRI revealed a semi-lobar HPE, atrophy of the vermis, partial agenesis of the corpus callosum, absence of occipital lobe and a large temporal and occipital fluid cavity communicating with the right ventricular junction (Fig. 1B). Both karyotype and Comparative Genomic Hybridization (CGH) Array detected no chromosomal abnormalities for both ll3 and ll5.

The homozygosity mapping identified 11 identical by descent regions larger than 1Mb, shared by the two affected siblings and heterozygous in the mother. Exome sequencing of the boy (ll5) and the mother (l2) revealed homozygous mutations in 7 genes located in these regions, among which 6 were on the same largest region of 18Mb located on 1p23 between rs230280 and rs12402927 (*CTRC*, *SPEN*, *WDR65*, *STIL*, *ORC1*, *LCCR7*), and 1 was located on 12q24 (*PGAM5*).

Frequencies of these mutations in public databases, bioinformatics predictions, conservation properties and physico-chemical gap between the wild-type and mutated amino acid were analyzed (Table 1). Regarding these elements, the 3 mutations presenting the most deleterious criteria were those located in *CTRC*, *ORC1* and *STIL*. The *CTRC* gene encoded the serine protease chymotrypsin C, a protein produced in small quantities by pancreatic cells that degrades trypsin. Mutations in *CTRC* have been associated with hereditary pancreatitis [16]. *CTRC* was not expressed in the mouse adult brain (Expression Atlas, European Molecular Biology Laboratory), and the only known link with development is its association with enamel development, the hard mineralized surface of teeth [17]. The combination of these elements permitted the *CTRC* mutation to be discarded. The *ORC1* mutation was intriguing because mutations in this gene were previously associated with Meier-Gorlin syndrome, a microcephalic primordial dwarfism [18]. *ORC1* encodes the subunit 1 of the origin recognition complex, a multi-subunit DNA binding complex, which is a key component of the DNA replication licensing machinery, and also plays a role in controlling centriole and centrosome copy number in human cells [19]. Except for microcephaly, the phenotype of Meier-Gorlin patients does not match with the phenotype of the affected siblings II3 and II5. In fact, the Meier-Gorlin syndrome has been characterized by a proportional short stature and microcephaly, with mean heights of approximately-5,5SD and-6SD at 5 and 12 years old [20]. Mean heights of II5 and II3 were-2SD and-2,5SD, which was significant but not major and did not correlate with their severe microcephaly. Moreover, the *ORC1* mutation found in the family was already described in the 1000 Genomes Project (1000G, April 2012), and predicted tolerated both by MetaSVM and MetaLR from ANNOVAR.

**Table 1:** Characteristics of homozygous candidate mutations.

Gene	Mutation	Amino acid conservation	Physico-chemical gap	Minor allele frequency		Bioinformatics predictions				
				1000G	ESP6500	Condel	SIFT	PolyPhen2	MetaSVM	MetaLR
CTRC	p.Glu96Lys	very conserved	low	MAF = 0,0005	no	deleterious	deleterious	probably damaging	deleterious	deleterious
SPEN	p.Arg672Gln	no data	low	no	no	neutral	tolerated	probably damaging	tolerated	tolerated
WDR65	p.Arg891Gln	poorly conserved	low	no	no	neutral	tolerated	benign	tolerated	tolerated
STIL	p.Gly717Glu	very conserved	high	no	no	deleterious	deleterious	probably damaging	deleterious	tolerated
ORC1	p.Arg728His	very conserved	low	MAF = 0,0005	no	deleterious	deleterious	probably damaging	tolerated	tolerated
LRRC7	p.Pro755Ser	moderately conserved	high	no	no	neutral	tolerated	benign	tolerated	tolerated
PGAM5	p.Arg118His	very conserved	low	MAF = 0,0005	MAF = 0,000308	deleterious	tolerated	possibly damaging	tolerated	tolerated

The amino acid conservation and physico-chemical gap come from Alamut software. Minor Allele Frequency (MAF) in the 1000 Genomes Project (1000G, April 2012), in the Exome Sequencing Project (ESP6500, October 2012) and bioinformatics predictions by SIFT, PolyPhen2, MetaSVM and MetaLR were performed using ANNOVAR.

The mutation that presented the most deleterious criteria was the *STIL* homozygous mutation c.2150G>A (p.Gly717Glu). Indeed, almost all bioinformatic tools used predicted that this mutation was likely to affect the protein function. This mutation was located in exon 12 of the gene *STIL* (or *SIL; SCL/TAL1 interrupting locus*) (RefSeqNG_012126.1), encoding a pericentriolar and centrosomal protein. It was absent in 1000G and the Exome Sequencing Project (ESP6500, October 2012) databases, and in the Genome Management Application (University of Miami Health System) that contains genomic data of about 200 Turkish families.

This mutation concerned a highly conserved nucleotide and amino acid through 12 species including *Tetraodon* and *Xenopus*, and the physico-chemical gap between Glycine and Glutamic acid is high.

Segregation analysis by Sanger sequencing in the 3 available family members (I2, ll3 and lI5) confirmed the recessive inheritance of the c.2150G>A (p.Gly717Glu) mutation in the *STIL* gene, with consistent genotypes (Fig. 1C).

*STIL* mutations have been described in rare cases of autosomal recessive primary microcephaly (MCPH) [21–23]. MCPH is a genetically heterogeneous disease characterized by an intellectual deficit and a pronounced reduction in brain volume, with or without architectonical anomalies, depending of the mutated gene. Eleven genes have been identified so far as being involved in the cause of this disease and most of these genes encode either centrosomal proteins or proteins associated with the poles of the mitotic spindle [24]. Several clinical and molecular genetic studies on microcephaly have been published. These studies have shown that in typical microcephaly, mutations in the ASPM gene were the most prevalent (14.1%) and mutations in *STIL* were less frequent (2.2%). They described several truncating mutations in the C terminus of the protein STIL (Fig. 1D) [21]. These truncating mutations deleted a motif involved in proteasomal degradation, called the KEN box, which would make STIL resistant to proteasomal degradation and cause centriole amplification. The mutation found in our HPE patients (p.Gly717Glu) was located in a conserved central part of the protein near a coiled-coil domain of the protein, but not in the regions interacting with the CPAP protein required for centriole assembly (Fig. 1D) [25].

Mouse embryos homozygous for the mutated *Stil* allele displayed multiple abnormalities, including forebrain midline defects and die by embryonic day (E) 10.5 [26]. These defects were reminiscent to a HPE phenotype and led Karkera *et al*. to look for an association with HPE and *STIL* mutations; however no causative mutations were noted in the 83 HPE patients studied [27]. We sequenced *STIL* in a series of 21 patients presenting HPE and microcephaly with 8 of them born from consanguineous parents, but we did not identify any mutations (data not shown). Among these patients, 12 have a European origin, 7 have a North-African origin, and 2 have a Middle-Eastern origin. Recently, two papers reported the implication of recessive *STIL* mutations, in a family with microcephaly associated with some midline defect [28], and in a consanguineous family with microcephaly and HPE [29] (Fig. 1D). Altogether, these results suggest that *STIL* complements the already long list of genes involved in HPE.

The *STIL* gene comprises 18 exons, and the protein was identified as being required for cell-cycle mitotic entry as well as for centriole formation and duplication [30]. Depletion of STIL blocks centriole duplication, while overexpression results in the generation of extra centrioles, an event known as centrosome amplification. These results suggest that the expression levels of STIL needs to be precisely controlled and this was achieved by proteasomal degradation during mitotic exit [25,30,31].

**Fig 2:**
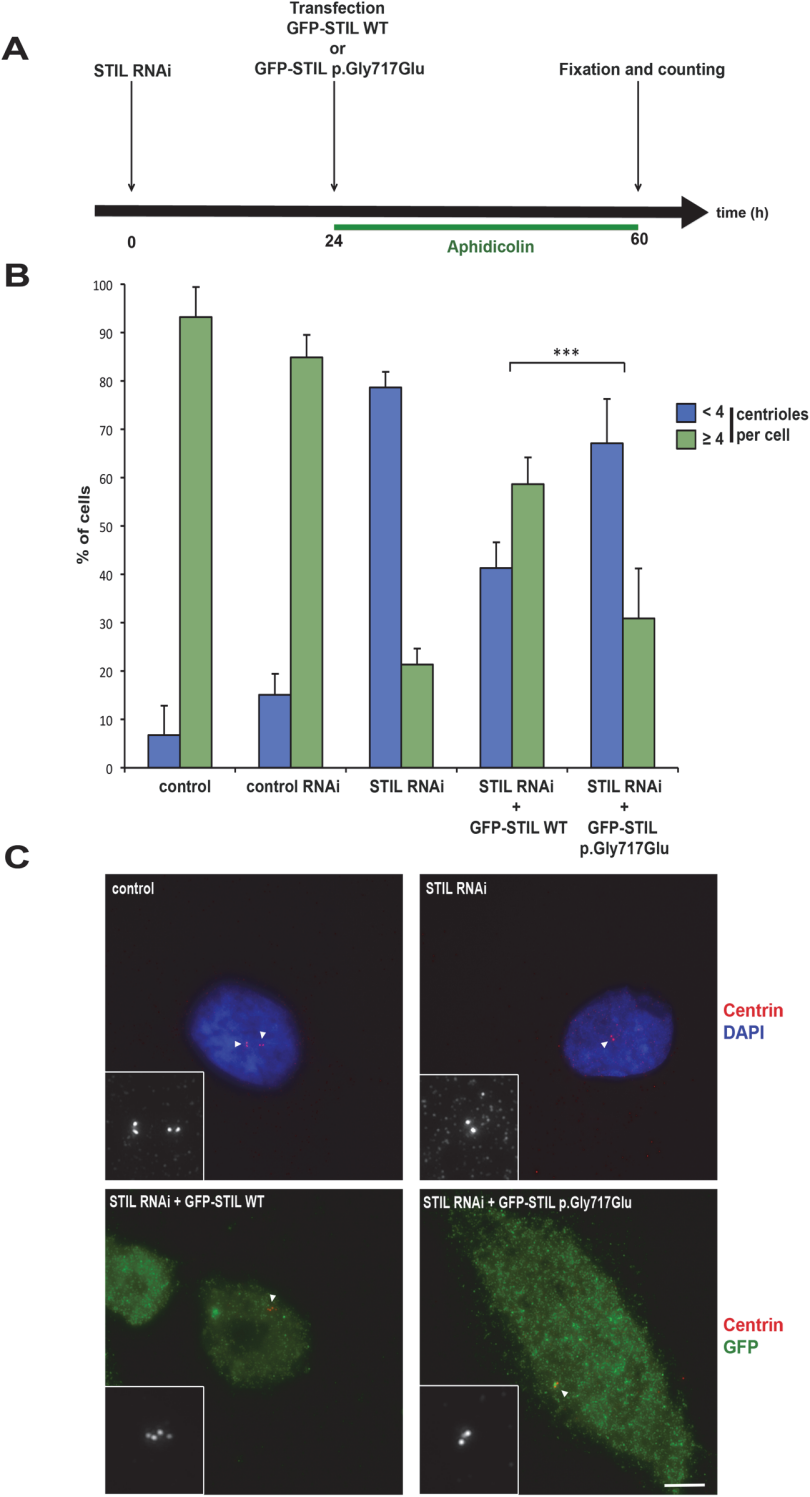
p.Gly717Glu cannot fully restore STIL depletion in synchronized U2OS cells. (A) Protocol used to assay the centriole duplication potential of WT and mutant STIL proteins. U2OS were treated with STIL RNAi, and transfected 24h later with GFP-STIL WT or GFP-STIL p.Gly717Glu constructs in aphidicolin containing medium (4 μg/ml). Cells were fixed and counted 36h later to allow centriole duplication. (B) Percentages of S phase cells containing <4 or ≥4 centrioles following control RNAi (scrambled) and STIL RNAi, followed or not by transfection with GFP-STIL WT or GFP-STIL p.Gly717Glu (p<0,001***). (C) Examples of S phase-arrested cells following different treatments. A control cell with 4 centrioles (top left panel), a STIL RNAi treated cell with 2 centrioles (top right panel), a STIL-depleted cell expressing GFP-STIL WT with 4 centrioles (bottom left), and a STIL-depleted cell expressing GFP-STIL p.Gly717Glu with 2 centrioles are displayed (bottom right). Centrin is shown in red (and in monochrome in the insets), DNA is blue (top panels) and GFP is green (bottom panels). The white arrowheads indicate the centriole region. The bar represents 10 μm.

In order to test the deleterious role of the *STIL* mutation p.Gly717Glu, we implemented transitory rescue experiments in U2OS cells (Fig. 2). Cells were first transfected with a siRNA targeting endogenous *STIL*, and 24h later with plasmids expressing RNAi resistant GFP-STIL WT or GFP-STIL p.Gly717Glu (Fig. 2A). Centrioles were counted 36h after transfection of GFP-STIL plasmids, allowing expression of the WT and the mutant GFP-tagged variants of STIL, and depletion of the endogenous STIL by RNAi. In order to have a homogeneous cell population, cells were synchronized in G1/S phase by aphidicolin treatment that blocked DNA synthesis but allowed centrioles to continue to replicate. In control U2OS cells, we observed approximately 93% of cells containing 4 centrioles and 7% contained less than 4 centrioles (Fig. 2B). As described in the literature [30], in absence of plasmid, the number of centrioles per cell decreased when cells were transfected with the *STIL* siRNA, with a majority of cells (80%) displaying less than 4 centrioles (Fig. 2B). When cells were transfected with GFP-STIL WT and siRNA, the GFP-STIL WT was able to rescue the duplication of centrioles, and after 36h, about 60% of cells had 4 or more centrioles. By contrast, when cells were transfected with siRNA and GFP-STIL p.Gly717Glu, the rescue was less efficient. Indeed, after 36h, 67% of the cells still had less than 4 centrioles (Fig. 2B) as illustrated in synchronized G1/S phase cells (Fig. 2C). However, we observed a partial rescue, suggesting a reduced but not null activity of STIL p.Gly717Glu on centriole duplication. The same result was observed in non-synchronized U2OS cells (S1 Fig.), suggesting a deleterious role of the mutation p.Gly717Glu for STIL in our patients.

The *Stil* gene, highly conserved among vertebrates, has been described to be widely expressed in the developing mouse embryo [26]. However no data is currently available about the early expression of *Stil* during vertebrate forebrain patterning. *In situ* hybridization (ISH) was used to determine whether the expression of *Stil* coincides with a regulatory function during ventral forebrain development when HPE arises.

**Fig 3:**
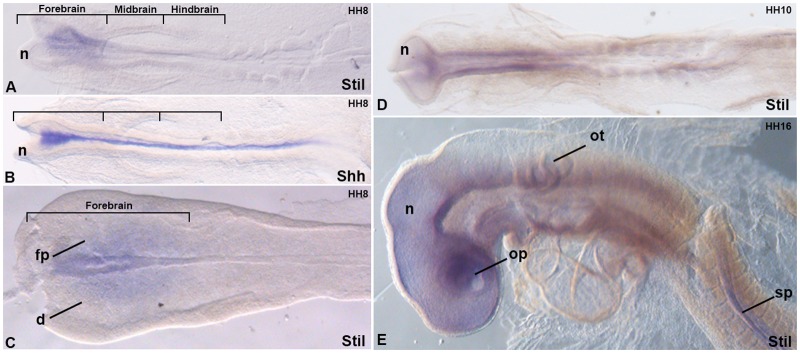
*Stil* was specifically expressed in the developing chick forebrain. *In situ* hybridization analysis of *Stil* (A, C-E) was performed on whole-mount chick embryos at developmental stages HH8, HH10 and HH16. Expression of *Shh* (B) was analyzed at HH8. A, B and D are dorsal views, E is a lateral view. (C) Flat-mounted preparation of the forebrain of the above embryo (A). Brackets indicate the compartments of the brain. d, dorsal border of the forebrain; fp, floor plate; n, neurectoderm; ot, otic vesicle; op optic vesicle, sp; spinal cord.

The first detailed description of *Stil* expression in the developing vertebrate forebrain has been presented here (Fig. 3A, C, D and E). *Stil* was expressed during the first stages of chick brain development (HH8) consistent with *Stil* having a role in ventral forebrain patterning (Fig. 3A). Significantly, the ventral view of the corresponding flat-mounted neural tube revealed the ventral neurectodermal surface specifically expressing *Stil* (Fig. 3C). *Stil* was expressed in the ventral forebrain in a domain that overlaps with the anterior expression domain of *Shh* (Fig. 3B), the main HPE gene [1]. Similarly, *Sufu*, a repressor of Shh pathway, was also expressed in this area [32]. Remarkably, *in vitro* experiments have shown that STIL interacts in the cytoplasm with SUFU to regulate Shh signaling [33–35]. This role may also be disturbed by the abnormal behavior of the mutated GFP-STIL (p.Gly717Glu) protein. Later between HH10 and HH16 (Fig. 3D, E), when patterning of the dorso-ventral forebrain has already initiated, *Stil* was expressed in all the neurectoderm tissue. When using a *Stil* sense probe as control, no staining was observed (data not shown). This expression study suggested that *STIL* may have a function during early patterning of the forebrain that could be linked to Shh signaling. An abnormal STIL protein would lead to a disturbed Shh signaling which could explain the HPE phenotype. Subsequently, the ubiquitous expression of *STIL* during brain growth could be responsible for microcephaly appearance through its implication in centriole formation [24].

This study confirms that homozygosity mapping associated with next generation sequencing in consanguineous families can be of great interest to identify new genes in heterogeneous pathologies. In fact, the results presented here confirm that mutations in *STIL* can cause HPE associated with microcephaly, with a recessive mode of inheritance. These findings have clinical application since this new gene will increase the panel of genes already tested for holoprosencephaly diagnosis.

## Supporting Information

S1 Fig GFP-STIL p.Gly717Glu failed to complement STIL-dependent centriole duplication in U2OS cells. U2OS cells were subjected to control RNAi and STIL RNAi together with expression of siRNA resistant GFP-STIL or GFP-STIL p.Gly717Glu for 48h. The cells were then fixed and stained for GFP (green), tubulin (blue) and centrin (red, and monochrome in the lower panels). (A) Control cell with 4 centrioles (left), STIL-depleted cell expressing GFP-STIL WT with 4 centrioles (middle), STIL-depleted cell expressing GFP-STIL p.Gly717Glu with 3 centrioles (right) during mitosis. Centrioles were indicated by the triangles and the insets show the centriole regions. Bar represents 10 μm. (B) Percentages of the interphase and mitotic cells containing 1, 2, 3, 4 or >4 centrioles following control RNAi (scrambled) and STIL RNAi with or without co-transfection with GFP-STIL WT or GFP-STIL p.Gly717Glu. The number of centriole was quantified in the GFP positive cells. Most STIL-depleted cells (70%) expressing GFP-STIL WT displayed 4 or more centrioles against only 30% for GFP-STIL p.Gly717Glu expressing cells (p<0,001***).
(TIF)
