# Supplementary figures and images for: Homozygous STIL Mutation Causes Holoprosencephaly and Microcephaly in Two Siblings
Source: PLoS One. 2015 Feb 6;10(2):e0117418. doi: 10.1371/journal.pone.0117418 (PMC4319975; doi:10.1371/journal.pone.0117418)

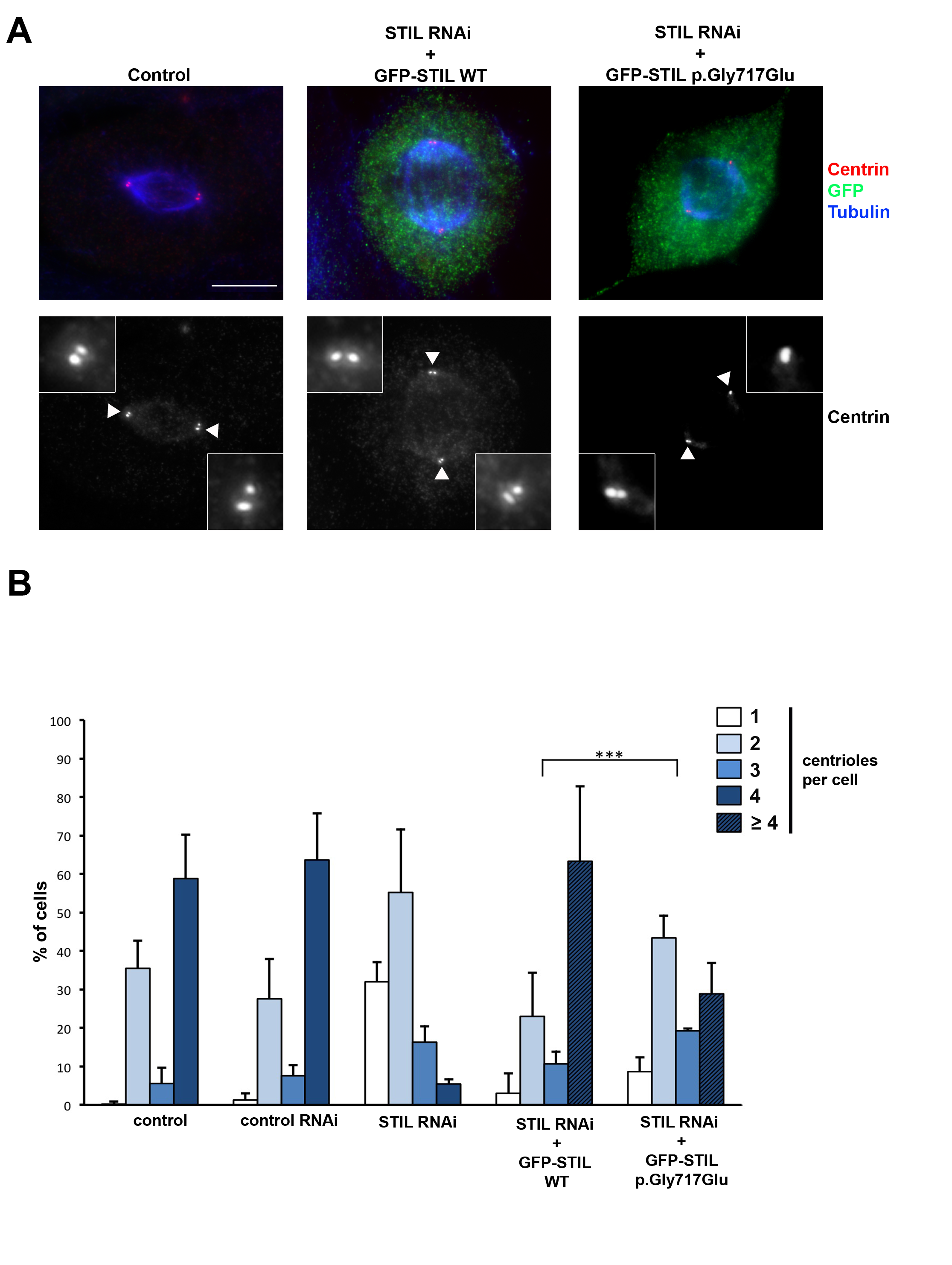

Supplement: S1 Fig — U2OS cells were subjected to control RNAi and STIL RNAi together with expression of siRNA resistant GFP-STIL or GFP-STIL p.Gly717Glu for 48h. The cells were then fixed and stained for GFP (green), tubulin (blue) and centrin (red, and monochrome in the lower panels). (A) Control cell with 4 centrioles (left), STIL-depleted cell expressing GFP-STIL WT with 4 centrioles (middle), STIL-depleted cell expressing GFP-STIL p.Gly717Glu with 3 centrioles (right) during mitosis. Centrioles were indicated by the triangles and the insets show the centriole regions. Bar represents 10 μm. (B) Percentages of the interphase and mitotic cells containing 1, 2, 3, 4 or >4 centrioles following control RNAi (scrambled) and STIL RNAi with or without co-transfection with GFP-STIL WT or GFP-STIL p.Gly717Glu. The number of centriole was quantified in the GFP positive cells. Most STIL-depleted cells (70%) expressing GFP-STIL WT displayed 4 or more centrioles against only 30% for GFP-STIL p.Gly717Glu expressing cells (p<0,001***). (TIF) [file pone.0117418.s001.tif]
